# Fermented Soy Products and Their Potential Health Benefits: A Review

**DOI:** 10.3390/microorganisms10081606

**Published:** 2022-08-09

**Authors:** Fernanda Guilherme do Prado, Maria Giovana Binder Pagnoncelli, Gilberto Vinícius de Melo Pereira, Susan Grace Karp, Carlos Ricardo Soccol

**Affiliations:** 1 Department of Bioprocess Engineering and Biotechnology, Federal University of Paraná (UFPR), Curitiba 81530-900, PR, Brazil; 2 Bioprocess Engineering and Biotechnology Department, Federal University of Technology-Paraná (UTFPR), Curitiba 80230-900, PR, Brazil

**Keywords:** oxidative stress, fermented soybean foods, bioactive compounds, genistein, daidzein

## Abstract

In the growing search for therapeutic strategies, there is an interest in foods containing natural antioxidants and other bioactive compounds capable of preventing or reversing pathogenic processes associated with metabolic disease. Fermentation has been used as a potent way of improving the properties of soybean and their components. Microbial metabolism is responsible for producing the β-glucosidase enzyme that converts glycosidic isoflavones into aglycones with higher biological activity in fermented soy products, in addition to several end-metabolites associated with human health development, including peptides, phenolic acids, fatty acids, vitamins, flavonoids, minerals, and organic acids. Thus, several products have emerged from soybean fermentation by fungi, bacteria, or a combination of both. This review covers the key biological characteristics of soy and fermented soy products, including natto, miso, tofu, douchi, sufu, cheonggukjang, doenjang, kanjang, meju, tempeh, thua-nao, kinema, hawaijar, and tungrymbai. The inclusion of these foods in the diet has been associated with the reduction of chronic diseases, with potential anticancer, anti-obesity, antidiabetic, anticholesterol, anti-inflammatory, and neuroprotective effects. These biological activities and the recently studied potential of fermented soybean molecules against SARS-CoV-2 are discussed. Finally, a patent landscape is presented to provide the state-of-the-art of the transfer of knowledge from the scientific sphere to the industrial application.

## 1. Introduction

Oxidative stress (OS) is related to biologically harmful impacts of free radicals presenting itself as an imbalance between oxidants and antioxidants. During normal metabolic and oxidation processes, free radicals are released and, when excessive in the body, they cause oxidative damage to proteins, lipids, DNA, and RNA. Their deleterious effects include the attack on healthy cells leading to cell death and causing chronic diseases and premature aging. Continuous oxidative damage prevents the activity of a biological system, weakening the body’s defense mechanism [1,2]. Thus, the balance of the operating system in the body has a critical role in the beginning and/or development of various pathologies. Dietary factors with oxidants and antioxidants are reported to be responsible for altering the operating system in the body and improving the host’s antioxidant defense system [3].

Chronic diseases such as cancer [4], cardiovascular diseases [5], neurodegenerative disorders [6], metabolic syndrome [7], and inflammatory diseases [8], are associated with oxidative stress. The prevention or delay of these disorders can be related to the consumption of products with potent antioxidants. Fermented foods, green and red teas [9], grape seed [10], broccoli [11], soybean [12], goat milk [13], common bean [14], and others are examples of foods containing antioxidant components. 

Correlated to a series of beneficial health effects, fermented foods appear as a promising alternative due to the presence of many compounds with bioactive properties including antioxidants. These promoting actions are credited to the metabolic and biotransformation activities carried out by microorganisms during the fermentation process [15]. Thus, the beneficial effects of eating fermented soybean foods on OS have been widely mentioned [16,17]. The main biological-associated components includes soybean isoflavones, flavonoids such as coumestans, non-flavonoids such as lignans, stilbenes, β-carotene, and anthocyanidins and polyphenols [18,19]. Compounds that have phytoestrogen and antioxidant functions. Several studies show the in vitro and in vivo antioxidant impacts of isoflavones, which directly suppress free radicals. The aglycone forms genistein and daidzein, found in greater quantities after fermentation, are mainly effective in suppressing reactive oxygen species (ROS) [17,20,21]. 

In addition to the antioxidant effects, other bioactive properties are conferred to fermented soy products, such as immunomodulatory, anticancer activity, prevention of cardiovascular diseases, the pathophysiology of Alzheimer’s disease, and cholesterol reduction [22,23,24,25]. The present review aims to provide an overview of the effect of soybean food fermentation and discuss its properties and performance of bioactive compounds.

## 2. Soybean

Soybean (*Glycine max*) is one of the most widely grown oilseed crops in the world and one of the cheapest and most abundant sources of vegetable protein consumed as a food and dietary supplement [26]. The soybean has in its composition about 40% protein, 20% lipids, 35% carbohydrates, 5% minerals, and 10% moisture, in addition to other compounds such as fatty acids, vitamins, flavonoids, isoflavones, phenolic acids, and saponins [27,28].

In the context between global supply and demand for food, soy stands out for being a highly nutritious grain capable of providing a range of by-products for human consumption, in addition to attributing an important role in the production of animal protein (chicken, pork, and beef) [29]. Of the world oilseed production, 60% corresponds to soy and, of this total, only 6% is consumed in the form of whole grains, whole grains, and fermented products. The remaining 94% is processed industrially, being transformed into oil for human consumption, production of biodiesel, and development of chemicals, food, and cosmetics (18–20% of this total). The remainder crushed is usually transformed into protein-rich bran used for animal feed [30].

**Figure 1:**
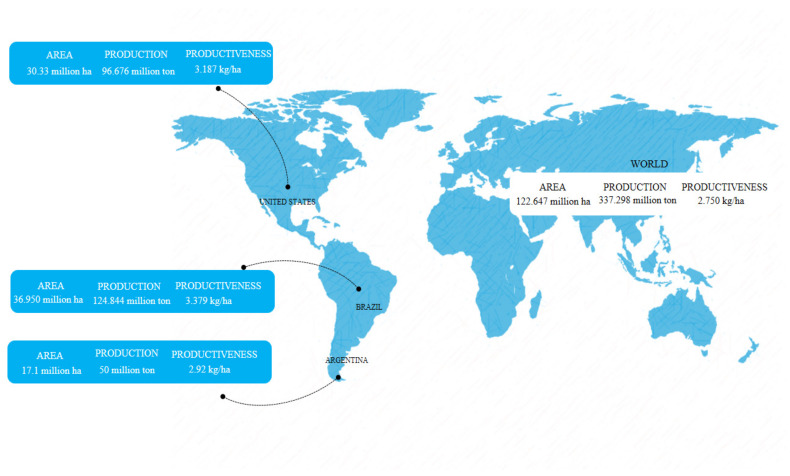
Production, productiveness, and total planted area in hectares of soybean in 2019/2020.

The world soybean production in the 2019/2020 harvest was estimated by the U.S. Department of Agriculture (USDA) at 337.298 million tons, in a total world area occupied by the cultivation of 122.647 million hectares, corresponding to the average productivity of 2.750 kg/ha [31]. The largest suppliers are Brazil (124.84 million tons), the United States (96.67 million tons), and Argentina (50 million tons), with a total planted area of 36.950, 30.33, and 17.1 million hectares, respectively. In Brazil, the average productivity was 3.379 kg/ha, while in the United States and Argentina the value was 3.187 kg/ha and 2.92 kg/ha, respectively (Figure 1) [31,32].

2020/2021 was considered the largest soy harvest in history, with an estimated world production of 362.85 million tons, which corresponds to an 8% increase compared to the previous harvest (Table 1). The projection still consists of an increase in exports, imports, animal, and domestic consumption. Thus, the estimated value of the final world stock fell by 2%, reaching 98.39 million tons [31].

USDA projects the 2021/22 world soybean crop at 372.56 million tons. Brazil remains the world’s largest producer; however, the forecast was cut from 144 million, in the previous bulletin, to 139 million tons. Brazilian ending stocks of soy were also reduced from 28.25 to 23.55 million tons but estimates for Brazilian soy exports were maintained at 94 million tons. Argentina’s production was also projected to fall by 46.5 million tons, a cut of 3 million from the previous report. For the US, the USDA brought a slight increase from 120.45 to 120.7 million tons [33].

Asian countries are characterized by high grain consumption. In recent years, due to increased demand and domestic supply, China has become the largest importer of soybeans, accounting for more than 60% of world soy trading. It is because Chinese production accounts for only 20% of domestic demand and a large amount of soy is needed to supply pork feed. Compared to other crops, the application of soy resources is potentially greater, taking the efficiency of land use as follows: a soybean yield of 3000 kg/ha becomes a product to produce 343 kg of beef, 600 kg of swine, or 1200 kg of birds. In the absence of soybean meal, production dropped to 250 kg of beef [34,35].

In Europe, soybean cultivation is mainly in the southern and eastern regions, but still plays a minor role due to the higher latitudes with relatively cool conditions [36]. One important reason for expanding soybean cultivation into central and northern growing areas is the high demand for soy protein in Europe, which would require 9–12% of its arable land to be sown to soybeans [37].

**Figure 2:**
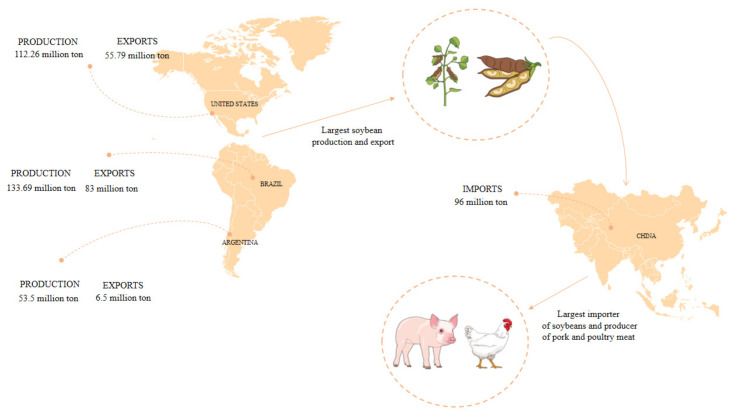
Soybean production and export of largest producers and estimate for imports in the 119 2020/2021 harvest.

In the 2020/2021 harvest, there is an estimate for Chinese imports of 96 million tons. Brazil also assumes the position of largest grain exporter, with more than 60% of the harvest destined for this purpose. Motivated by the high Chinese demand and the percentage, Brazilian exports are expected to reach 83 million tons. The global export forecast is 312.80 million tons, of which 55.8 million tons are from the United States and only 6.5 million tons from Argentina (Figure 2) [31].

Plant genotype, location, climate, water, and maturity group are responsible for the quality characteristics of soy, e.g., protein, oil, fatty acids, soluble sugars, and isoflavones. The grain consists of 8% husks, 90% cotyledons, and 2% hypocotyls. The protein composition of the soybean grain on a dry basis is on average 40% (Table 2). The storage of proteins occurs in the intermediate layers of the grain, cotyledons, and hypocotyls. In the shell, the outer layer, there are greater amounts of carbohydrates cellulosic material [38,39].

In addition to the high protein content, the soybean contains other bioactive and remarkably rich components, such as isoflavones, anthocyanins, and saponins. They are compounds known to have antioxidant capacity that is related to different health health benefits [40,41].

## 3. Fermented Soybean Products

**Figure 3:**
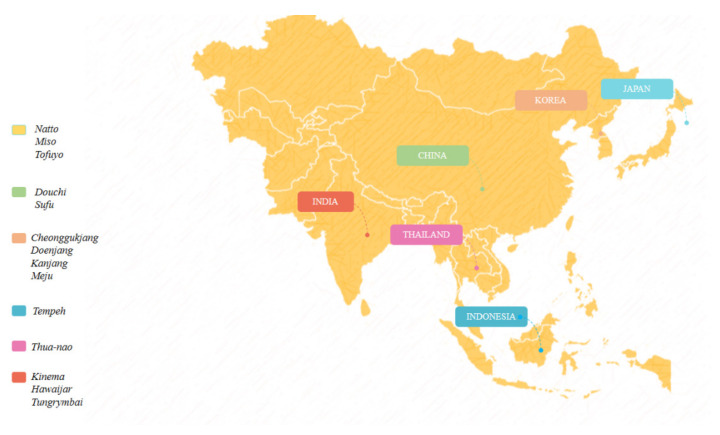
Products fermented soy in Asian countries.

The production and consumption of fermented soy are widespread in Asian countries. The main soybean products include natto, miso, tofuyo (Japan), douchi, sufu (China), cheonggukjang, doenjang, kanjang and meju (Korea), tempeh (Indonesia), thua-nao (Thailand), kinema, hawaijar, tungrymbai (India) (Figure 3), in addition to other widely consumed products, such as sauce, pasta, and soybean milk [42]. Records of some production methods were found in Cheminyoshul, a Chinese manuscript dating back to 530–550 dC, and others in the Korean manuscript Samkuksaki, dating from the 1392s, pointing to the consumption of fermented soybeans since the 12th century [43].

The large consumption of soybeans in Asian countries is related to the widespread adoption of grain cultivation. The variety of appropriate climates and geographic regions resulted in highly sizable crops, making soybean a staple in the region. With insufficient meat consumption, these fermented foods played a vital role as a source of protein in the Asian diet [44,45].

In ancient times, the basic idea behind soybean fermentation was the preservation of food. In the current perspective, the research is interested and directed to the application of fermentation to improve the bioactive components of soybean, responsible for health benefits, and reduce anti-nutritional factors [46,47].

The difference between fermented soybeans is based on several parameters, but mainly due to the microorganism used in the process. Thus, fermented soybean products are different in terms of aroma, texture, and therapeutic and nutraceutical values. Some fermentations occur only with bacteria, others using only filamentous fungi, and, in many cases, both these microbial groups are used. Some products are fermented only with Bacillus (natto, kinema, chungkookjang); some are fermented with fungi *Aspergillus oryzae*, *Mucor* spp. *Rhizopus* spp. and *Fusarium* spp. (douchi, tempeh, miso, tofu) and in some cases both microorganisms are used, as in the case of doenjang, where the bacteria involved in this process would be *B. subtilis* and fungi include *Rhizopus* spp., *Mucor* spp., *Geotrichum* spp., and *Aspergillus* spp. [42,48,49,50,51].

Soybean protein and isoflavones are the main functional constituents of fermented soybean foods. Soybean consists of one of the plant sources with the highest abundance of isoflavones. Because their chemical structure is similar and has an affinity for estrogen receptors, these compounds are usually called phytoestrogens [52,53].

The native forms of isoflavones have their bioavailability compromised because they are usually combined with sugars that minimize their absorption through the human intestinal tract. Isoflavones are categorized into two groups: glycosides and aglycone. The beneficial and functional effects of isoflavones on health are conferred to their aglycone forms, which are absorbed more quickly by the human body. In unfermented soybean, the presence of aglycone isoflavones is 2–3% of the total composition—this content being mainly corresponded to β-glucoside isoflavones [54,55,56].

Thus, the biotransformation of glycosidic forms into aglycones through fermentation is a desirable process to increase and produce more biologically active forms. In fermented soy products, the aglycone values vary from 40 to 100%. The conversion of the glycoside into isoflavone aglycones occurs through the action of β-glycosidase produced by microorganisms during the fermentation process. In addition to a higher absorption rate, aglycone forms have greater antioxidant activity than glycosidic forms. This explains the fact that the consumption of fermented soybean products in Asian countries is associated with the reduction of chronic diseases since the consumption of natural antioxidants is efficient in reducing the harmful impacts of reactive oxygen species (ROS) and in adjusting the body’s antioxidant load [57,58,59].

Soy protein fraction has many inhibitory enzymes, such as proteinase and trypsin, which make them less digestible. During fermentation, proteolytic enzymes generated by microbial populations hydrolyze proteins into peptides and free amino acids responsible for antioxidant activity and increasing the digestibility of soybean protein [60,61].

In addition to proteins and isoflavones, soybean is made up of numerous other functional and nutritional substances, such as fatty acids, vitamins, peptides, minerals, flavonoids, phenolic acids, and saponins [62].

Natto consists of a popular and traditional food in Japan that has been consumed since the 17th century and produced by fermenting soybeans cooked with strains of *Bacillus subtilis* var natto. It is known to have large amounts of peptides because, in the fermentation process, proteins are cleaved by extracellular proteases produced by the *Bacillus* strain, which increases the free amino acid content by 10% to 30%. Studies report that the proteins derived from this food consist of at least seventeen different amino acids, including glutamic acid, glutamine, aspartic acid, leucine, proline, serine, lysine, methionine, threonine, glycine, isoleucine, tyrosine, phenylalanine, histidine, arginine, alanine, and valine [63,64]. The consumption of natto has been shown to have an anti-aging effect, prolonging life expectancy, due to the metabolites found in natto extracts, for example, the enzyme nattokinase. Often, reduced life expectancy is caused by oxidative stress, and the relatively high antioxidant activities of natto are mentioned [65].

Miso is a fermented soybean paste, prepared from steamed soy, salt, and koji—cooked cereal or soy malted with *Aspergillus oryzae*. It is a traditional Japanese spice used to add flavor to soups and dishes consumed at breakfast by most Japanese families for over 1000 years. The process of maturation of the Miso takes from three to twenty-four months and involves different microorganisms, such as molds, yeasts and, lactic acid bacteria, which act by hydrolyzing the components of [66,67]. There are different types of miso, which vary according to local traditions and available ingredients; and this food can be classified according to the koji used: (1) rice miso, made by adding rice koji to soy; (2) barley miso with the addition of barley koji to soy and soy miso, made only with soybean. It is reported that during the miso ripening process, the peptides formed are made up of 3–20 amino acids and still include amino acids such as glutamic acid, aspartic acid, and proline [68,69].

Tofu is a Japanese fermented soy curd similar to cream cheese—a characteristic that results from the ripening or maturation process by proteases, carbohydrases, and other catabolic enzymes found in red koji (*Monascus fungus*) or koji yellow (*Aspergillus oryzae*), used in the preparation of this food together with tofu (vegetable cheese based on soybean) [50,70]. Its functional properties were investigated and associated with the presence of bioactive peptides. Thus, tofu came to be seen not only as a nutritional accompaniment but becoming a valuable source of protein [71,72].

Douchi is a popular product consumed for at least 2000 years by the Chinese, as a source of protein and flavoring ingredient. The preparation of the douchi is carried out in two stages: pre-fermentation, which consists of an aerobic process using several microorganisms as the starter culture (for example, *Aspergillus oryzae*, *Zygosaccharomyces rouxii*, *Lactobacillus plantarum*, *Bacillus subtilis*) and takes 12–15 days; and post-fermentation, where the addition and mixing of salt and other spices is carried out and left for 9 months in anaerobic fermentation, a process where the development of the special nutrients and flavor of the douchi occurs [73,74]. In recent years, douchi has attracted attention as a functional food source. Some studies have revealed the benefits of this food to health, including antioxidants, antihypertensive activity and even lowering blood pressure [75,76].

Mentioned as “Chinese cheese” due to its texture, sufu is a traditional fermented soy product that has been used as a flavor enhancer and appetizer. There are different types of sufu, which are produced by various processes in different locations in China through microbial fermentation, and based on the types of starter culture; sufu can be classified into fungi fermented sufu (inoculated with *Actinomucor*, *Mucor* or *Rhizopus*), sufu fermented by bacteria (inoculated with *Bacillus* or *Micrococcus*), and others (naturally inoculated) [77,78].

## 4. Nutritional Changes in Fermented Soybean Products

Fermentation enriches the nutritional value of foods by increasing the content of vitamins, essential amino acids, or fatty acids, allowing detoxification and removal of anti-nutritional factors. In addition to proteins and isoflavones, soybean is made up of numerous other functional and nutritional substances, with increased fermentation process through microbial biotransformations. Microorganisms with abilities to produce specific hydrolytic enzymes, such as protease, amylase, and β-glucosidase, play a fundamental role in increasing functional properties [79,80,81].

The modification of isoflavones occurs through β-glucose enzymes, which degrade cellulose, hydrolyze the β-d-cellulose terminal non-reducing glucoside bond, and, consequently, release β-d-glucose. Thus, the amount of glycosides decreases by hydration by β-glucosidase, increasing the amount of isoflavones aglycones [82,83]. After ingesting the aglycone isoflavones, such as genistein and daidzein, they are absorbed by the blood vessels, hydrolyzed in the small and large intestine by intestinal hydrolytic enzymes and microbial glycosidases through deglycosylation, increasing their bioactive potential [84].

Some final characteristics of the fermented product, as well as the changes that occur during soy fermentation, are related to the type of microorganism used. In tempeh, for example, several amylases, lipases, and proteases are produced by fungi of the genus *Rhizopus* spp. These hydrolyze macronutrients into simpler, water-soluble compounds, resulting in the production of vitamins, phytochemicals, and antioxidant constituents [1]. The increased antioxidant effect of tempeh can still be attributed to the increased levels of polyphenols released by cell wall degradation by enzymes secreted by *Rhizopus* fungi during preparation in the boiling stage and through the course of fermentation [85].

Due to the metabolic activity of starter cultures, the levels of vitamin B complexes are also increased during fermentation. In tempeh, *Rhizopus* and the bacteria *K. pneumoniae* and *C. freundii* are the main producers of vitamin B_12_. In natto, *Bacillus* is the agent responsible for the increase of viitamin K2 [86,87]. The functionality of these vitamins is well known, being essential bioactive substances that act in the coordination of the nervous system and the development of the brain. Some studies still confirm the significant increase in gamma-aminobutyric acid (GABA) in fermented soy products, responsible for the regulation of the central nervous system [88].

Microbial proteolytic enzymes involved in the fermentation hydrolyze protein content into peptides. The length of the chain and the composition and sequence of amino acids interfere with the biological activity of the peptide and, during enzymatic hydrolysis in fermentation and digestion, inactive bioactive peptides are released. Furthermore, the bioconversion of high molecular weight proteins into minors increases the solubility [89,90]. One of the main biochemical changes that occurs during fermentation is the hydrolysis of proteins by microbial proteases and the enrichment of nutritional effects depends on this reaction [91].

Angiotensin-converting enzyme (ACE) inhibitory peptides are generated by proteolytic degradation of glycinin and β-conglycinin, which consists of protein fractions from soybean. This enzyme acts in the conversion of angiotensin I into angiotensin II and inactivation of the bradykinin vasodilator, raising blood pressure and the risk of cardiovascular disease. Hydrophobic amino acids (Try, Phe, Trp, Ala, Ile, Val, and Met) or positively charged amino acids (Arg and Lys) show greater affinity with ACE. There are three classifications for ACE-inhibiting peptides, being (1) true inhibitor, unaffected by gastrointestinal digestion, (2) substrate, converted into other peptides with less activity in gastrointestinal digestion, and (3) prodrug, converted to true inhibitors by gastrointestinal digestion [92,93]. Still, several other nutritional changes are reported as a consequence of the soybean fermentation process, such as the increase in total soluble iron, the level of folic acid, the composition of tocopherol, with the levels of beta-, gamma- and delta-tocopherol being increased [40]. 

In summary, many metabolic activities and biotransformations take place during the soybean fermentation process. There are several beneficial health effects of the final fermented product, and its consumption is related to a series of bioactivities that will be mentioned below.

## 5. Potential Beneficial Health Effect of Soybean Fermented Product

### 5.1. Antioxidant Effect

Many normal reactions in the body form by-products such as free radicals, which are species with unpaired electrons. If the antioxidant defense systems are not efficient, there is an increase in tissue damage and oxidative stress, associated with cell apoptosis and the appearance of several chronic diseases [61].

Due to the beneficial effects of the prevention of these diseases caused by cellular oxidative processes and reactive oxygen species, antioxidants, essential to prevent the formation and suppress the activities of reactive nitrogen and oxygen species, become the main compounds with benefits for health to be included in the diet [94].

Several fermented soybean foods have bioactive components, such as polyphenols, phenolic acid, saponins, sterol, and flavonoids, that protect against oxidative damage, with flavonoids and phenolics being fundamental compounds responsible for antioxidant activity [95].

Phytoestrogenic compounds and phenolic compounds are an important class of phytochemical antioxidants. In soybean and soybean products, aglycone forms are characterized by having greater estrogenic and antioxidant potential [96].

### 5.2. Anticancer Effect

Environmental factors, especially diet, are considered to play a key role in carcinogenesis. The incidence of cancer in the Asian population is relatively low, and Asians traditionally consume large amounts of soy-based foods, which are rich in isoflavones [97]. One of the first studies that linked cancer risk reduction and the consumption of fermented soy-based foods took place in Singapore in 1991, reporting that a soy-rich diet resulted in less breast cancer development in women in pre-menopause [98].

Soybean isoflavones are believed to have the potential to reduce cancer risk through their antioxidant activity and estrogen-like structure. Genistein presents estrogen binding affinity compared to estradiol (estrogen steroid hormone) receptors ER-α and ER-β of 4% and 87%, respectively. As a result, it binds to these receptors and plays an important role in preventing hormone-related cancers [99]. In addition, genistein is a known tyrosine kinase inhibitor and acts by preventing topoisomerase and angiogenesis. Through these functions, their effect is evident in the cascades of proliferation signals. Some bioactive peptides from soybean can also prevent the growth of tumor cells, such as lunasin and saponins, repelling the formation of the cell membrane and promoting cell apoptosis [100,101].

Several soybean products are mentioned for their anti-cancer potential. In Korea, fungi and *Bacillus* sp. are used in the fermentation process in Meju, a dry soybean block, which is used to produce other products such as Kanjang, Doenjang, and Gochuchang. The anti-cancer potential of Doenjang is associated with compounds, such as trypsin inhibitor, isoflavones, vitamin E, and an unsaturated fatty acid that contributes to the biological effect. In addition, Doenjang extracts invigorate glutathione S-transferase and increase the vitalization of natural killer cells [102].

Mostly, the anticancer effects of fermented soy products are associated with isoflavones. Some studies have shown that methylation-mediated epigenetic gene silencing can be reversed. Genistein is said to have a broad-spectrum anticancer effect on cancers of the breast, prostate, esophagus, pancreas, stomach, and colon, and metacarcinoma, lymphoma, and neuroblastoma. It also acts as a positive regulator of the mRNA expression of several tumor suppressor genes, counteracting the function of growth-stimulating factors and inhibiting cell malignancy. Therefore, the consumption and inclusion of fermented soybean foods have stood out as a new therapy for the treatment of tumors [103,104].

### 5.3. Anti-Obesity and Antidiabetic Effect

Physiologically, obesity consists of an imbalance between energy intake and consumption that indicates an excessive accumulation of fat in the tissue and is considered a major health problem that is advancing significantly worldwide. Obesity is generally correlated with diabetes and metabolic syndromes leading to hyperinsulinemia and dyslipidemia [105].

As a result, interest in combating obesity and overweight is growing. Several studies show that a change in the diet prevents and alleviates a series of metabolic imbalances characterized by central obesity, dyslipidemia, and high fasting glucose. Some benefits are attributed to the physiologically active components of certain foods, therefore, they are used to prevent obesity and its complications [106,107].

The isoflavones daidzein and genistein found in high levels in fermented soybean foods are mentioned as having bioactivity, regulating the generation of lipids and thermogenesis in vivo. Through lipogenesis (synthesis of fatty acids and triglycerides), hyperlipidemia (high levels of fat particles in the blood), hyperglycemia (elevated blood glucose), and improved insulin resistance, aglycones and metabolites demonstrate their anti-obesity effects [108]. Some studies also associate these effects with the bioactive phytochemical content of fermented soybean foods as alpha-amylase and alpha-glucosidase inhibitors, protease inhibitors, hemagglutinin, and crude fibers, able to disturb normal metabolism and assist in the management of obesity and different metabolic disorders [109].

The consumption of isoflavones is also associated with an increase in HDL cholesterol and a reduction in total cholesterol, LDL, and triglycerides. In addition to isoflavones, soy proteins, as well as peptides, are active ingredients that lower the levels of LDL cholesterol and triacylglycerols [100].

There is an association between obesity and the transition from pre-menopause to post-menopause in women. This phase is correlated with the risk of several diseases due to the lack of hormonal regulation, including the accumulation of abdominal fat, hypertriglyceridemia, and high levels of low-density lipoprotein cholesterol (LDL-C), reduced high-density lipoprotein cholesterol (HDL-C), elevated blood pressure (BP), and impaired glucose tolerance/diabetes. Studies show that the consumption of fermented soybean foods rich in isoflavones has beneficial effects on the distribution of body fat and lipid profile in women during the menopause period, due to the structural similarity of these compounds with estrogen, their greater affinity for estrogen receptors and circulating concentration in the human body [110,111].

Clinical and experimental studies indicate that the population with obesity and overweight is more vulnerable to type 2 diabetes mellitus (DM2), with obesity dramatically increasing the likelihood of DM2. Individuals with type 2 diabetes mellitus are at increased risk of cardiovascular disease, even with aggressive control of glucose, cholesterol, and blood pressure [112,113].

Oxidative stress (OS) is closely associated with obesity and diabetes. Free fatty acids, which at high levels influence the production of reactive oxygen species (ROS) through mitochondrial electron transport chain complexes and enzymes in endothelial cells, decrease the bioactivity of nitric oxide, activate pro-inflammatory signaling pathways causing damage to cellular proteins and organelles. Damaged, more oxidizing mitochondrial enzymes enhance oxidative stress and cellular dysfunction. Chronic exposure to ROS negatively affects insulin signaling when stress pathways are activated. As a result, insulin resistance, glucose intolerance, β-cell, and mitochondrial dysfunctions are developed, advancing to a state of diabetes [114,115]. The consumption of foods rich in isoflavones is seen as a promising strategy in the treatment of diabetes and obesity. Genistein reduces the inflammatory state in obese people, decreases production, and neutralizes the effects of ROS, resulting in the relief of insulin resistance and, consequently, decreasing the risk of diabetes [116]. After ingestion, genistein enriches insulin resistance by increasing the production of insulin receptor substrate (IRS) 1, glucose transporter (GLUT) type 1, and N-terminal c-jun kinase, increasing the activity of superoxide dismutase, decreasing mitochondrial damage and lipid peroxidation. Daidzein, which is also found in soybeans, activates GLUT4 and IRS1 in adipocytes, adding insulin-stimulated glucose uptake [117].

Spermidine is a naturally occurring polyamine present in all living cells noted to play an important role in cellular functions and is found in different concentrations in fermented soy foods such as Chunjang (1.4–12.8 mg/kg), Doubanjiang (0.18 mg/kg), Douchi (74.92 mg/kg), and Sufu (1.3–32.87 mg/kg) [45,118].

Increased spermidine flux is associated with increased glucose and lipid metabolism. Many in vivo studies reveal that spermidine overexpression protects against diet-induced obesity, and an epidemiological study shows that foods rich in polyamines, such as spermidine, are associated with a lower occurrence of cardiovascular disease (CVD), corroborating the theory that spermidine is beneficial in the treatment of obesity [119,120,121].

### 5.4. Anti-Inflammatory Effect

Inflammation is a natural biological mechanism in the human body in which the immune system protects against tissue damage due to physical trauma, harmful chemicals, and microbial agents. A wide range of progressive diseases is related to inflammation, resulting in (i) dysregulation of cell signaling, (ii) exaggerated appearance of cytokines, (iii) abrogation of the barrier function to inflammatory cells, and (iv) oxidative damage of tissues and organs. Thus, some studies suggest that the reduction or inhibition of chronic inflammatory mechanisms can prevent numerous diseases. Thus, a diet with anti-inflammatory components has beneficial biological activities [122,123].

In the inflammatory reaction, the macrophage produces nitric oxide (NO), which is usually detected as iNOS (inducible nitric oxide synthase) [124,125]. Isoflavones act as inhibitors of NO production and, consequently, cancel the production of IL-1β and TNF-α pro-inflammatory cytokines. After ingestion, these compounds inhibit the expression of COX-2, production of pro-inflammatory cytokines, and activation of the nuclear transcription factor kappa-B (NF-κB). Thus, the expression of several genes during inflammatory responses is controlled, and regulation of innate and adaptive immunity occurs. Isoflavones still affect the mechanisms of inflammation containing the inflammatory process through different intracellular signaling pathways triggered by AP-1, PPAR, Nrf2, MAPKs [123,126].

Epidemiological investigations show the associations between different soy foods and inflammatory markers, including highly sensitive C-reactive protein (hs-CRP), interleukin IL-6 and IL-18. The high levels of intake of these foods, including miso and soy sauce, are related to a reduction in the serum level of IL-6, a pro-inflammatory cytokine associated with several chronic diseases [127].

Above all, the anti-inflammatory effects of isoflavones are confirmed by the fact that they act in the elimination of reactive oxygen species (ROS), which are directly involved in inflammation.

### 5.5. Preventive Effect against Cardiovascular Disease

Some evidence reports the association between high soy consumption and the preventive effect against cardiovascular diseases (CVD), such as a lower risk of ischemic heart disease (IHD) or stroke. Soy protein and isoflavones are the constituents responsible for the lower risk of CVD, in addition to their beneficial effects such as lipid profile, arterial stiffness, blood pressure, and endothelial functions [128].

Nattokinase (NK) is an enzyme contained in the sticky component of natto, cheese-like food made from soybeans fermented with *Bacillus* subtilis, which can dissolve thrombi and fibrin. Because it is considered stable in the gastrointestinal tract, NK becomes an appropriate agent for oral thrombolytic therapy [129]. It is because NK acts directly degrading fibrin or activates other fibrinolytic enzymes, such as pro-urokinase and tissue plasminogen activator (t-PA). NK also inactivates plasminogen activator inhibitor-1 (PAI-1) in vitro, the primary inhibitor of t-PA, resulting in the enhancement of fibrinolysis [130,131].

The development of intravascular thrombi causes a variety of CVDs. Studies suggest that natto has broad thrombolytic efficacy and its ingestion has protective effects against CVD [132].

### 5.6. Neuroprotective Effect

The human brain is singularly vulnerable to oxidative damage and has high oxygen consumption, in addition to having a relatively high content of polyunsaturated fatty acids (PUFA), which are sensitive to oxidation. Otherwise, neurons are particularly sensitive to disturbances in the balance between antioxidants and the production of reactive oxygen species (ROS), since the levels of antioxidant defense in the brain are negligible. High content of active redox metals is found in the brain, which promotes the formation of ROS and is associated with the development of pathologies [133].

A possible therapeutic approach for the treatment of neurodegenerative diseases is to control microglial activation and reduce the number of pro-inflammatory factors since the overproduction of inflammatory mediators and cytokines causes chronic neuroinflammation, develops several neurodegenerative diseases, and can occasionally lead to neural cell death [134].

Studies show that isoflavones are protective against neuronal cell death, elevate existing neuronal function, and boost neuronal regeneration. Thus, interest in the consumption of fermented soybean foods rich in isoflavones is growing due to their supposed beneficial effects, such as the ability of genistein to inhibit the apoptotic signaling cascade in neurons [135].

### 5.7. Anti-Aging Effect

Aging is seen as an inevitable, universal, multifactorial, and complex progressive decline in the physiological functions of all living beings, affecting the condition of relative stability and making it susceptible to age-related injuries and diseases [136,137].

Therapies that help achieve healthy aging have become an efficient path to longevity in humans. In this search for the longevity of the body, antioxidant therapy has beneficial effects, pointing out the role of dietary antioxidants. The accumulation of oxidized molecules, such as lipid peroxides, proteins, and damaged DNA mediated by oxidative stress (OS), is the result of the aging process and the administration of antioxidants can prevent oxidation or exclude the production of free radicals, characterized by affecting the rate of aging [108,138,139].

Traditionally, fermented soybean food products are mentioned as having anti-aging properties. These effects are associated with the isoflavones aglycone genistein and daidzein [140]. Studies suggest the anti-aging effect of Tempeh in the pre and postmenopausal with the maintenance of the quality of the uterus, the improvement of skin quality, and bone strength [99,141]. It is because isoflavones act in the replacement of estrogen, improving the quality of life of postmenopausal women [142].

Some research show that neuroinflammation is related to low-grade systemic inflammation, common in aging. The cascade of neuroinflammation also correlated with systemic inflammation is one of the most widely accepted suspicions regarding Alzheimer’s disease (AD), one of the main common forms of age-related dementia. Antioxidant and anti-inflammatory nutrients are mentioned as agents that help to reduce or delay the development of AD [143].

Neuroinflammation in the brain can be reduced by the isoflavones present in fermented soy foods, known to have antioxidant activity. A promoter of proinflammatory activity, IL-1β, is decreased while a potent anti-inflammatory cytokine, IL-10, is increased. Moreover, the intake of isoflavones increases cognitive capacity and prevents oxidative damage in neurons. In AD neurodegeneration, the most damaging sequel is memory loss, with the first implicit mechanism being the deficit of cholinergic neurons, where the transmission of information is canceled due to the lack of neurotransmitters such as acetylcholine (ACh). Research suggests that isoflavones reverse amnesia by increasing acetylcholine and reducing levels of acetylcholinesterase [144,145,146].

After dietary supplementation with Chungkookjang, a fermented soybean paste from Korea cultivated by *Bacillus* sp., components, such as the isoflavonoids daidzein and genistein, elevated the activity of superoxide dismutase, an important free radical scavenging enzyme [147]. It has also been reported that, by improving memory functions and other neurological indications, gerbils induced by stroke are improved [148]. These, express cerebral ischemia after transitory artery occlusion, and afterward, have global neural cell death due to the addition of oxidative stress, and neuroinflammation [149,150].

Oxidative stress is the cause of reduced life expectancy. Some findings suggest that Natto significantly prolonged the life of nematodes, increased resilience to oxidative stress, and postponed the accumulation of lipofuscin, a characteristic of aging cells. This is cited for its anti-aging effect, relying on actions such as preventing heart attacks, stroke, osteoporosis, bowel disease, and improved cognitive function, especially with age [128,151].

The anti-aging effect of fermented soy foods can also be attributed to the high concentration of spermidine, bioactive polyamines found in high levels in foods such as natto and tempeh. Such a compound has several important functional and regulatory properties related to the physiology of cell aging, such as reversing memory loss, improving the blood lipid profile, and reducing cardiovascular risks, inducing autophagy in damaged cells [152].

## 6. SARS-CoV-2

A new strain of Coronavirus not previously identified in humans was reported in Wuhan, China in December 2019, being identified as a beta type of Coronavirus ß-CoV Group 2B. A total of seven human coronaviruses (HCoVs) have been identified before: HCoV-229E, HCoV-OC43, HCoV-NL63, HCoV-HKU1, SARS-CoV—responsible for severe acute respiratory syndrome, MERS-COV—responsible for the Middle East respiratory syndrome, and recently the new coronavirus, called SARS-CoV-2—responsible for causing the new severe respiratory inflammatory disease, COVID-19 [153,154].

As of early May 2022, a total of 514,918,067 confirmed cases and 6,240,940 deaths caused by COVID-19 have been reported to the World Health Organization (WHO) [155]. SARS-CoV-2 enters the body using angiotensin-converting enzyme 2 (ACE2) and transmembrane protease, serine 2 (TMPRSS2), as target receptors to infect the cells. After affecting the epithelial cells of the human respiratory tract, the rapid replication of the virus leads to a storm of pro-inflammatory cytokines and chemokines. This hyperinflammatory state causes oxidative stress leading to damage to the alveolar and endothelial cells of the lung and chronic lung inflammation [156,157].

Given the pandemic scenario, numerous researchers investigate risk factors, clinical manifestations, and possible preventive and therapeutic actions. Health conditions such as obesity, diabetes, previous morbidities with risk of immunodeficiency, and chronic cardiovascular, renal, and respiratory diseases are also investigated for association and relationship to the high severity of COVID-19 [158]. Among the complications caused by the disease are ARDS, septic shock, coagulation dysfunction, metabolic acidosis, cardiac arrhythmia, kidney damage, liver dysfunction, heart failure, or secondary infection [159].

A therapeutic strategy for the control of SARS-CoV-2 consists of identifying anti-inflammatory agents to act on the reduction of uncontrolled inflammation in patients and the receptors for the ACE-2 enzyme since it is widely expressed by epithelial cells of the lung, kidney, heart, blood vessels, and intestine [160]. Flavonoid-derived bioactive compounds, such as isoflavones, are mentioned for their significant health benefits such as antibacterial, antioxidant, anticancer, anti-inflammatory, and immunomodulatory bioactivity [161].

There are a few studies related to dietary habits as a risk factor for COVID-19 instability. However, some differences in diet have been hypothesized to play a potential role in disease and fatality rate variability [162]. Based on mechanistic and clinical data, vitamins and folate, polysaccharides and dietary fiber, lipids, peptides, and natural polyphenols are known to be necessary for the body’s immune system against viruses [163]. 

For example, certain countries, such as Bulgaria, Greece, Romania, and Turkey, where there is high consumption of some types of fermented foods (cabbage and milk), are associated with lower mortality rates. The possible protective effects of antioxidants and angiotensin-converting enzyme (ACE) inhibiting peptides present in fermented foods may justify this hypothesis [164,165].

Adem et al. [166] performed a molecular docking study to identify the ability of 80 flavonoid compounds to bind to the 3-chymotrypsin-like protease (3CLpro), a known enzyme important for SARS-CoV replication. Other polyphenols and flavonoids, such as daidzein and genistein (found in fermented soy products), have been proposed as potential inhibitors of the main SARS-CoV-2 protease [167].

As already mentioned, bioactive peptides with therapeutic properties, including antihypertensive antioxidant, antitumor, and antidiabetic, are present in fermented soy products. Fermented soy peptides have previously demonstrated activity against several viruses, including the SARS-CoV responsible for the SARS outbreak in 2003 [168]. In the soybean fermentation process, the proteolytic degradation of the soy protein fractions (glycine and β-conglycinin) generates the ACE inhibitory peptides [169]. Studies have already mentioned the identification of ACE inhibitory and antihypertensive peptides in Natto and, also, two ACE inhibitory peptides isolated from tofu [71,170]. Other foods, such as douchi (fermented by *A. egyptiacus*) and sufu (a soybean fermented by the fungus), have peptides with ACE inhibitory activity [171,172].

Oba et al. [173] conducted a study with Natto to investigate the antiviral activities of this food against SARS-CoV-2. The results showed that Natto extract fully inhibited severe acute respiratory syndrome coronavirus 2 (SARS-CoV-2) infection in the cells. The protease activities of Natto extract were able to proteolytically degrade the British variant of the spike protein (receptor binding domain; RBD) of SARS-CoV-2, resulting in the inhibition of viral infections in cells.

A study conducted by Chourasia et al. [168] from soy cheese fermented with *Lactobacillus delbrueckii* WS4, identified and selected peptides for antiviral activity in silico. A total of twenty-three peptide sequences were examined for binding affinity to critical residues of the SARS-CoV-2 RBD protein and important catalytic residues of the SARS-CoV-2 pro-enzyme 3CL using molecular docking. The authors also showed in molecular docking studies of the selected peptides that they revealed a potential peptide “KFVPKQPNMIL”. This peptide showed a strong affinity for significant amino acid residues, for host cells (RBD) of the SARS-CoV-2 peak S1 glycoprotein that are responsible for binding the virus to the human ACE2 protein receptor and also an affinity for the important viral proteolytic enzyme 3CLpro. for viral replication.

Therefore, it can be concluded that fermented soy cheese could be explored as a prophylactic food for SARS-CoV-2 and related viruses. Furthermore, the multi-target inhibitor peptide, which effectively inhibited both viral proteins, could be used for in vitro and in vivo functions against SARS-CoV-2.

## 7. Bioactive Compounds in Soybean Fermented Product

Soy has long been consumed as a health food, and fermented soybean products are important components of traditional diets in Asian countries. The benefits of fermented soy are attributed to its phytochemical content and bioactive compounds, which confer numerous benefits to human health [174].

Many compounds are responsible for the bioactive properties of fermented foods mentioned in several studies. Isoflavones are compounds found in tempeh, which act as antioxidants and are also related to many chronic diseases [175]. Surya and Romulo showed that tempeh extracts protect HepG2 cells (human liver cancer cell line) against induced oxidative stress by reducing ROS generation, and eventually cell death [176].

Chungkookjang has compounds such as isoflavone aglycones, peptides, and dietary fiber, and is rich in poly-γ-glutamic acid (γ-PGA). Consumption of this food can act on memory impairment induced by Alzheimer’s disease and cerebral ischemia, so it prevents and alleviates neural cell survival, thus improving brain insulin sensitivity and neuroinflammation [147].

To investigate the hypothesis that chungkookjang consumption improves sensitivity and insulin secretion capacity, an animal model study reminiscent of the characteristics of type 2 diabetes in Asians was conducted. A high concentration of daidzein was observed and related to the anti-diabetic properties of chungkookjang, capable of improving glucose regulation by potentiating insulin secretion and reducing insulin resistance [177].

Recently, research in Japan has shown that higher consumption of natto and miso is associated with a lower risk of mortality [178]. These foods are sources of bioactive compounds such as nattokinase, bacillopeptidase F, vitamin K2, dipicolinic acid, γ-polyglutamic acid, isoflavones, vanillic acid and syringic acid, which have health-promoting effects [54].

Nattokinase is shown to be responsible for anti-thrombotic and anti-coagulative activities. The anti-thrombotic effect of NK can be used for the treatment of cardiovascular diseases, and such a compound also acts on amyloid degradation related to Alzheimer’s disease and on the suppression of atherosclerosis, heart attack, and stroke in sick patients [179,180]. The health benefits of miso are associated with the presence of isoflavones, such as 8-OH-daidzein, 8-OH-genistein, 6-OH-daidzein, which have strong antioxidant activity, this is related to a series of beneficial effects on human health [181].

Doenjang is an important food consumed in Korea, such food shows strong activities against several carcinogens/mutagenic agents, such as aflatoxin B1. Park et al. showed through studies that genistein and linoleic acid present in doenjang extracts have strong antimutagenic activities, being more effective among the other bioactive compounds found in this food, such as β-sitosterol, soy saponin, α-tocopherol, genistein, and phytic acid [182].

Fermented soy foods are composed of molecules, vitamins, and peptides that are found in greater availability after the fermentation process, which point to being a potential source of numerous health benefits. The bioactive compounds found in fermented soy products are associated with the microorganism used in the process as well as the traditional practice used in each region.

Several bioactive compounds are found in different fermented soy products, these and their health benefits are illustrated in Table 3.

**Table 3:** Bioactive compounds of different fermented soy products and their health benefits.

Soybean Products	Bioactive Compounds	Health Benefits	References
Tempeh	Isoflavone aglycone	Antioxidant properties	[183]
Tempeh	Isoflavone aglycone	Protection of HepG2 cells from oxidative stress	[176]
Tempeh	Genistein	Immunomodulatory Function	[184]
Tempeh	Trans-cinnamic acid	Antioxidant properties	[59]
Chungkookjang	Poly-γ-glutamic acid (γ-PGA)	Prevention of memory loss from Alzheimer’s and cerebral ischemia	[147]
Chungkookjang	Daidzein	Anti-diabetic property	[177]
Chungkookjang	Poly-γ-glutamic acid	Anti-obesity effect	[185]
Chungkookjang	Bacillomycin D and surfactin	Antimicrobial activity	[186]
Natto	Nattokinase (NK)	Anti-thrombotic and anti-coagulant activities	[179]
Natto	Vitamin K2	Reducing osteoporotic fracture risk	[187]
Natto	Bacillopeptidase F	Anti-thrombotic and blood pressure-lowering	[188]
Natto	Nattokinase (NK)	Fibrinolytic activity	[189]
Miso	Isoflavones aglycones	Anti-tumoral activity	[190]
Miso	Isoflavones aglycones	Protective effects against stroke	[191]
Miso	Isoflavones aglycones	Sympathetic nerve activity	[192]
Doenjang	Linoleic acid and Genistein	Antimutagenic active	[182]
Doenjang	Genistein	Antimutagenic and anticancer activities	[193]
Doenjang	Genistein	Anti-obesity effects	[194]
Kinema	Poly-γ-glutamic acid (γ-PGA)	Suppression of post prandial hyperglycemia	[195]
Kinema	Isoflavones aglycones	Antioxidant properties	[42]
Kinema	Group B saponins	Prevention of dietary hypercholesterolemia	[196]
Douchi	β-glucosidase and protease	Antioxidant activity	[197]
Sufu	Isoflavones aglycones	Enhancement of the physiological function	[198]

## 8. Recent Patents and Innovations on Bioactive Compounds in Soybean Fermented Product

As presented throughout this review, the bioactive compounds present in fermented soy products are related to several beneficial activities for human health and well-being. Scientific studies confirm this potential, and to complement this view, a patent search was conducted showing recent advances and innovations in the use of such compounds.

The patent search was conducted on the Derwent Innovations Index patent database, on 25 March 2022, performing one search for each bioactive compound in the field “Title”, namely *Linoleic acid*, *Daidzein*, *Genistein*, *Isoflavone aglycone*, *Nattokinase*, *Cinnamic acid*, and *Vitamin K2*, using the wildcard * to retrieve all documents containing the defined word roots. The keywords were combined with the International Patent Classification (IPC) A61P (Specific therapeutic activity of chemical compounds or medicinal preparations) using the Boolean operator AND. The IPCs A61P 003/04 (Anorexiants; Antiobesity agents), A61P 003/10 (Hyperglycaemia, e.g., antidiabetics), A61P 009/12 (Antihypertensives), and A61P 037/04 (Immunostimulants) were used to refine the search when necessary [199]. The time interval was the last five years, 2018 to 2022.

**Figure 4:**
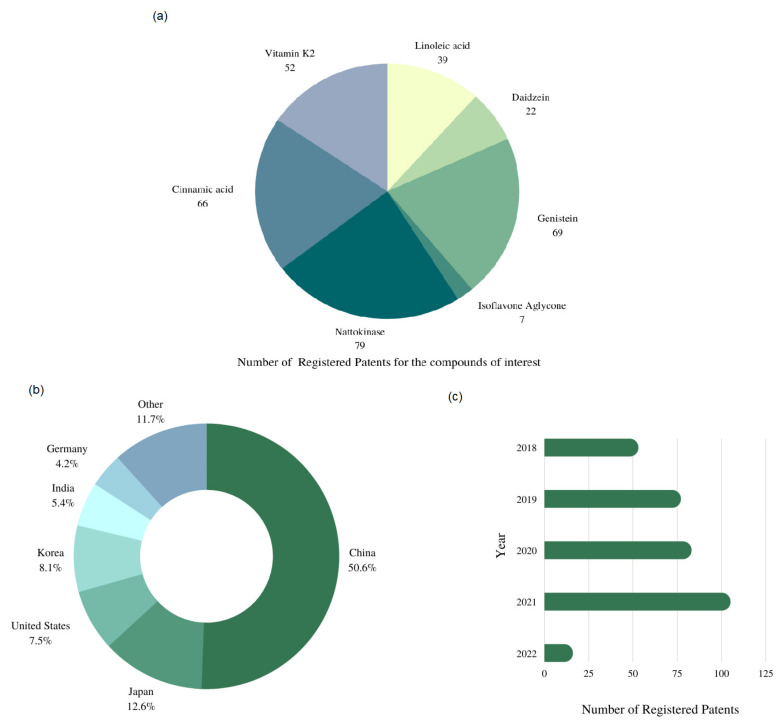
Number of registered patents for the compounds of interest (**a**), countries (**b**), and years (**c**) of publications.

After analyzing the documents by reading the titles and abstracts, 334 documents were classified and analyzed using Microsoft Excel software, California, USA. Along these years it is possible to observe an increase in the number of registered patent documents related to the development and innovation of products made from substances obtained in the soybean fermentation process. The apparent decrease in the number of documents in 2022 is attributed to the date of search (March 2022) and to the secrecy period of usually 18 months before publication (Figure 4c). China (CN) and Japan (JP) were the countries that filed the most patents (Figure 4). China accounted for 169 registered patents, representing 50.6% of the total analyzed, while Japan registered 42 patents (12.57%) (Figure 4b).

The substances generally most abundant in the fermented soybean product were nattokinase, genistein, and cinnamic acid because they are widely used in pharmaceutic formulations, for instance, in the prevention and treatment of chronic diseases. This was observed in terms of technology, because the highest number of documents was found for nattokinase (79), followed by genistein (69) and cinnamic acid (66) (Figure 4a).

The main company (assignee) that contributed to these patent filings was Hughes Biotechnology Co., Ltd.^®^, Taipei City, Taiwan, with 11.59% patent documents. This company is specialized in the development and manufacturing of plant-based nutraceuticals, focusing on the discovery and development of dietary ingredients that are based on the most current science, and reformulating existing ingredients to increase their potency. Other important assignees were Dongguan Anhao Pharm Co., Ltd.^®^ Dongguan, China, with 10.6% documents, a skin health management company that integrates product technology research and development, sales, and service, and Kobayashi Pharmaceutical Co., Ltd.^®^, Osaka, Japan, with 7.5% documents, that develops ideas for pharmaceuticals and various other applications in daily life, such as dental hygiene skincare, and nutritional supplementation.

## 9. Conclusions

It can be concluded that the future of fermented foods is quite optimistic since consumer awareness of natural sources is growing with a health-promoting effect. In the interest of preventive and therapeutic strategies, it is worth considering the potential of fermented foods and their bioactive compounds capable of reversing or preventing the pathogenic processes associated with metabolic diseases. Natural antioxidants are found as one of the main components with beneficial effects in the prevention of many diseases caused by cellular oxidative processes and reactive oxygen species; oxidative stress (OS) is closely associated with a series of chronic diseases and metabolic imbalance. In this context, interest arises in fermented soy foods, which have bioactive compounds such as flavonoids, isoflavone, peptides, and soy proteins. Soy aglucone isoflavones are antioxidant compounds and their activities are associated with the ability to eliminate reactive oxygen species (ROS). The study carried out with fermented soy milk represents a new strategy for researching peptide-based therapeutics against SARS-CoV-2 and related viruses, investigating the inhibitory action of peptides derived from this product and other fermented soy products on responsible protein molecules by entry into the host cell and viral replication.

## Figures and Tables

**Table 1:** Estimated global supply and demand for soybeans in the 2020/2021 harvest (in millions of tons).

Initial Stock	Production	Export	Import	Animal Consumption	Domestic Consumption	Final Stock
100.27	362.76	312.80	158.02	360.73	161.93	98.39
−11%	8%	4%	3%	4%	5%	−2%

**Table 2:** Nutritional composition of soybean.

Components	% Grain	Chemical Composition (% Dry Weight)			
		Proteins	Lipids	Carbohydrates	Others *
Husks	8	9	1	86	4.3
Hypocotyls	2	41	11	43	4.4
Cotyledons	90	43	23	29	5.0
Total	100	40	20	35	5.0

* Minerals, vitamins, phytates, and isoflavones.
